# Plasmonic Effects of Au@Ag Nanoparticles in Buffer and Active Layers of Polymer Solar Cells for Efficiency Enhancement

**DOI:** 10.3390/ma15165472

**Published:** 2022-08-09

**Authors:** Muheeb Ahmad Alkhalayfeh, Azlan Abdul Aziz, Mohd Zamir Pakhuruddin, Khadijah Mohammedsaleh M. Katubi

**Affiliations:** 1 School of Physics, Universiti Sains Malaysia, Minden 11800, Penang, Malaysia; 2 Institute of Nano Optoelectronics Research and Technology (INOR), Universiti Sains Malaysia (USM), Gelugor 11800, Penang, Malaysia; 3 Department of Chemistry, College of Science, Princess Nourah Bint Abdulrahman University, P.O. Box 84428, Riyadh 11671, Saudi Arabia

**Keywords:** Au@Ag NPs, polymer solar cell, PHJ PSCs, plasmonic effect, volume ratios NPs, plasmonic effect

## Abstract

Embedding nanoparticles (NPs) in the buffer layer of bulk heterojunction polymer solar cells (BHJ PSCs) excites the surface plasmonic polaritons and enhances the pathlength of the light in the solar cells. On the other hand, embedding NPs in the active layer significantly improves absorption and increases the production of electron-hole (e-h) pairs in BHJ PSCs. Increasing the volume ratio of NPs embedded in BHJ PSCs enables the direct interfacing of the NPs with the active layer, which then serves as a charge recombination center. Therefore, this study integrates the aforementioned phenomena by exploiting the effects of embedding plasmonic Au@Ag NPs in the buffer and active layers of PSC and then determining the optimum volume ratio of Au@Ag NPs. The results show the absorption is increased across the 350–750 nm wavelength region, and the PCE of the device with embedded Au@Ag in two locations is enhanced from 2.50 to 4.24%, which implies a 69.6% improvement in the PCE in comparison to the reference cell. This improvement is contributed by the combined localized surface plasmon resonance (LSPR) effects of multi-positional Au@Ag NPs, spiky durian-shaped morphology of Au@Ag NPs, and optimized volume ratio of Au@Ag NPs embedded in the PEDOT: PSS and PTB7:PC_71_BM layers.

## 1. Introduction

Polymer solar cells (PSCs) are typically materials appended with a light-absorbing or active layer that is usually composed of a blend of a fullerene (acceptor) and a conjugated polymer (donor). PSCs have garnered the attention of researchers in recent years due to their (i) low temperature and solution requirement for synthesis, (ii) relatively low cost of fabrication, (iii) low density, and (iv) compatibility with flexible substrates [1,2,3,4,5,6,7,8]. In addition, PSCs fabricated from π-conjugated carbon-based semiconductors have demonstrated immense potential for the fabrication of Internet of Things (IoT) devices or sensors [9,10,11,12,13,14]. PSCs are particularly appealing, since they do not emit toxic materials or greenhouse gases and need little maintenance during operation. The constant need for energy, as well as the limited use of its current primary sources (petroleum, natural gas, and coal), with their negative long-term environmental repercussions, mandates the fast development of clean and renewable energy sources. Solar energy is regarded to be the only sustainable form of electric power capable of saving our environment from pollution.

Even with recent advances, PSCs exhibit relatively lower power conversion efficiency (PCE) compared to their inorganic counterparts because of their intrinsically short exciton diffusion length, low carrier mobility, and restricted absorption of incident light, which all give rise to low internal quantum efficiency. Various attempts have been exercised to improve their PCE that have led to the formation of several mechanisms, such as surface modifications or functionalizations, used to increase the ability of the thin active layer to absorb light through periodic grating structures in electrodes, modifying the structural configuration of the layers, reconstructing the structure of optical devices for improved light distribution, and the inclusion of metallic nanoparticles (MNPs). Among the above strategies, MNPs have garnered broad interests as an effective way to trap light in the active layer, increase the dissociation of excitons, and improve the light absorption without increasing the thickness of the active layer due to their near-field coupling effect or localized surface plasmon resonance (LSPR) [15,16,17,18,19,20,21,22,23,24].

Gold (Au) and silver (Ag) are the most well-known options in the field of plasmonics due to their higher free-electron densities, good stability, and their resonance across the solar spectrum range [25,26,27,28,29]. The inclusion of Au and/or Ag into PSCs has been carried out in numerous studies. Earlier, Gao et al. embedded Au NPs between indium–tin–oxide (ITO) and a buffer layer (poly(3,4-ethylenedioxythiophene)): poly(styrenesulfonate) (PEDOT: PSS) to improve PCE from 2.81 to 3.25%. Nonetheless, these embedded Au NPs between ITO and a buffer layer resulted in carrier recombination, thereby reducing the PCE of PSCs [30]. Gao et al. embedded cooperative plasmonic Au:Ag NPs in the MoO_3_ buffer layer, which increased the PCE from 2.98 to 3.32%, which is contributed by the LSPR effect of Au:Ag NPs [31]. Rezaei et al. improved PCE in PSCs from 1.16 to 2.33% by embedding Au NPs with a graphene shell inserted between the buffer layer and active layer [32]. However, the LSPR effect may decline with an increase in the distance between MNPs and the active layer [33]. To enhance the PCE, it is essential to improve the broadband absorption of the active layer.

Hybrid metal nanostructures, bimetallic, core–shell, clustered, and mixed MNPs have been embedded in the active layer to enhance absorption in a broad wavelength range [34,35,36,37,38,39,40]. Kacus et al. improved PCE from 2.11 to 3.29% by embedding Au:Ag NPs into the active layer of PSCs, which infers a 55% enhancement relative to the reference cell [41]. Shaban et al. enhanced the inherent feature of optical absorption by embedding Ag NPs into the active layer. The study reported an increase in the short-circuit current density (J_sc_) from 19.7 to 26.7 mA/cm^2^ [42]. Nonetheless, by incorporating MNPs into the active layer, the MNPs may possibly become charge recombination sites that initiate exciton quenching and non-radiative energy interaction between the metal NPs’ organic and photo-active layers [43,44,45]. To prevent recombination of this carrier and enable a direct interface between MNPs and the active layer, studies have attempted to cover bare MNPs with dielectric materials such as TiO_2_ and SiO_2_ [46,47,48,49]. The constraint, however, is the negative effect of dielectric materials that leads to a reduction in the LSPR effects. In this research, the appropriate volume ratios (amount) of MNPs required to be embedded in the active layer serve a significant function in improving the efficiency of PSCs. Furthermore, this study applies an approach that incorporates the plasmonic nanostructures simultaneously in diverse sites to enhance broadband light absorption effectively. Inside, the active layer can be used to increase the absorption of incoming light in the active layer without increasing its thickness. This approach can enhance the possibility of photon absorption, even in ultra-thin layers.

## 2. Experimental Section

### 2.1. Materials

**Figure 1:**
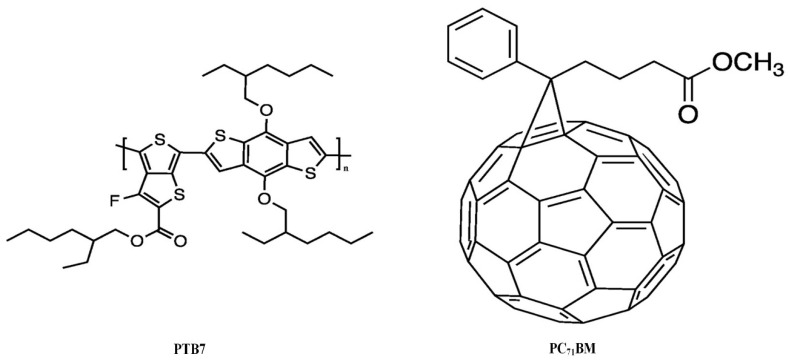
Structures of the active layer of BHJ PSCs consists of PTB7 and PC_71_BM.

The active layer of bulk heterojunction (BHJ) PSCs is composed of [4,8bis[(2ethylhexyl)oxy]-benzo[1,2-b:4,5-b’] dithiophene-2,6-diyl][3fluoro2[(2ethyl-hexyl) carbonyl]-thieno-[3,4-b] thiophenediyl (PTB7), and [6,6]-Phenyl (C71) butyric acid methyl ester, and a blend of isomers (PC71BM) (Figure 1). First, 10 mg/mL of PTB7 and 15 mg/mL of PC71BM were dissolved using 3% and 97% of 1,8-diiodooctane and chlorobenzene, respectively, and subsequently stirred all night in a glove box. PEDOT:PSS was utilized as a hole transport layer due to its facile processing, long-term stability, high transparency in the visible range, and harmless nature. Additionally, ITO was employed as an anode layer on account of its high optical transmittance and low resistivity (15–25 Ω/sq) features. The ITO electrode was etched off at specific portions of the glass slide using hydrochloric acid (HCl) and zinc metal (Zn). Afterwards, the ITO glass was washed with ethanol, acetone, isopropyl alcohol, and ultra-pure deionized (DI) water. Au@Ag NPs were synthesized from the following precursor materials: gold chloride trihydrate (HAuCl_4_.3H_2_O), ascorbic acid, silver nitrate (AgNO_3_), and polyvinylpyrrolidone (PVP). The entire materials were procured from Sigma-Aldrich (St. Louis, MO, USA).

### 2.2. Synthesis of Core–Shell Au@Ag NPs

Ag formed a complex with Au in an aqueous composite solution to fabricate core–shell Au@Ag durian-shaped NPs. Next, 20 µL and 200 µL of 10 mM concentrations of AgNO_3_ and HAuCl_4_, respectively, were mixed with 10 mL DI water to produce the precursor solution. The resultant solution was stirred for 20 s after introducing the mixed, freshly prepared ascorbic acid (100 mM) with molar ratios of 40 µL. The successful synthesis of the core–shell Au@Ag NPs is indicated by a color change in the mix solution to blue. Next, 2 mg of PVP dissolved in 80 µL of water was subsequently added to improve the stability of the solution.

### 2.3. Fabrication of BHJ PSCs

**Figure 2:**
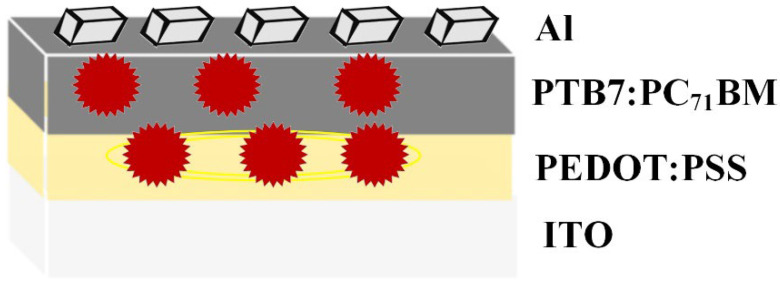
Device architecture of BHJ PSCs with embedded Au@Ag NPs in two different layers.

PEDOT:PSS embedded with Au@Ag NPs was spin coated at 3500 rpm for 30 s to form a thin layer (40–60 nm) on ITO-coated glass substrates. The volume ratio of Au@Ag NPs to PEDOT: PSS was maintained at 14%, according to our prior study [50]. The sample was subjected to annealing at 80 °C for 120 min to enhance the adsorption of PEDOT:PSS film to ITO substrate. Subsequently, PTB7:PC71BM embedded with varying volume ratios of Au@Ag NPs (2%, 4%, 6%, 8%, and 10% to PTB7:PC71BM) was deposited on glass/ITO/PEDOT:PSS + Au@Ag NPs (14%) via spin coating at 1100 rpm for 30 s to obtain a 100 nm-thick layer. Lastly, a 100 nm aluminum (Al) electrode was evaporated using the conventional thermal evaporation method at a pressure of 4 × 10^−5^ mbar. The BHJ PSCs embedded with plasmonic NPs at the buffer layer (PEDOT: PSS) and active layer (PTB7:PC71BM) were graphically illustrated in Figure 2.

### 2.4. Measurements

A UV-Vis spectrophotometer was used to determine the absorption spectra of ITO/PEDOT:PSS+Au@Ag NPs/PBT7:PC71BM+Au@Ag NPs. Transmission electron microscopy (TEM) was utilized for the detection of the dimension and profile of the synthesized NPs. The morphologies of the PTB7:PC71BM with and without Au@Ag NPs were studied using atomic force microscopy (AFM). The electrical properties of BHJ PSCs were characterized with and without Au@Ag NPs in two dissimilar layers, and were measured using a solar simulator (with a white LED source), a computer connected to the equipment, and Labview software (Forter Transient Measurement System). The power output of the white LED was 100 mW/cm^2^ (1-Sun condition). The short-circuit current density (J_sc_), open-circuit voltage (V_oc_), fill factor (FF), and PCE of the solar cells were derived from the electrical characterizations.

## 3. Results and Discussion

**Figure 3:**
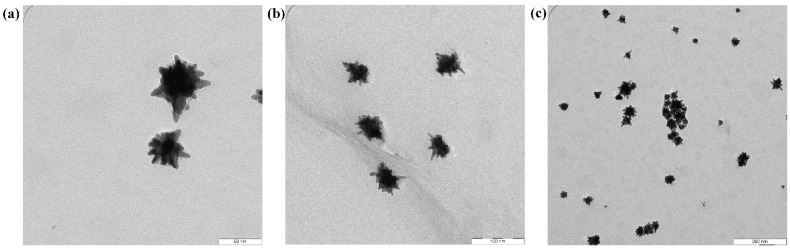
TEM images of Au@Ag NPs with (**a**) 50 nm scale, (**b**) 100 nm scale, and (**c**) 200 nm scale.

**Figure 4:**
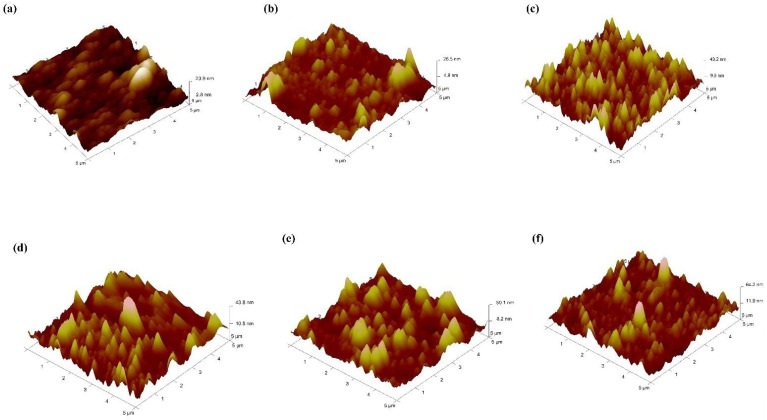
AFM images of the active layer (**a**) without NPs, (**b**) with Au@Ag NPs-2%, (**c**) with Au@Ag NPs-4%, (**d**) with Au@Ag NPs-6%, (**e**) with Au@Ag NPs-8%, and (**f**) with Au@Ag NPs-10%.

The durian-shaped core–shell Au@Ag NPs were synthesized based on our earlier study [16]. From the TEM images (Figure 3), the particle sizes of Au@Ag NPs are approximately 50–55 nm. The observed dark color of the cores (Figure 3a) confirmed the successful growth of Ag NPs on the Au NPs surface. Furthermore, all the Au@Ag NPs sizes showed compatibility with the PEDOT: PSS and PTB7:PC71BM film. Figure 4 presents 3D AFM images of the active layer (PTB7:PC71BM) embedded by varying volume ratios of Au@Ag NPs deposited on the PEDOT: PSS+14% Au@Ag NPs film. A random distribution of bowl-shaped nano-holes is discernible in each sample. Additionally, all films have different sizes, surface roughness, and form. The variations in the embossed feature for all the samples are due to the varying volume ratios of PTB7:PC71BM-to-Au@Ag NPs. The sharp spiked Au@Ag NPs on the PTB7:PC71BM exhibited higher curvature and height than the reference cell. Furthermore, the root mean square (RMS) of roughness increased concomitantly with the volume ratio of PTB7:PC71BM-to-Au@Ag NPs. The RMS roughness of the reference cell (ITO/PEDOT:PSS/PTB7:PC71BM) without the NPs is 5.7, but increases to 6.0, 8.3, 8.4, 12.0, and 12.7 nm for the cells embedded with NPs at diverse sites (active and buffer layer). It can be deduced that the RMS roughness values of samples embedded with Au@Ag NPs volume ratios of 8% and 10% are larger compared to those of the other samples, which can be attributable to the increase in height (z-axis) resulting from an increase in the volume ratio of NPs in the active layer. The surface roughness generated by these NPs allows light to be trapped within the hybrid active layer, allowing the light to stay in the active layer for a longer period of time. Furthermore, the alloyed Au@Ag NPs in the PTB7:PC71BM layer have grown horizontally and vertically. Large NPs aggregation has been detected in some areas of the coated film, suggesting that series resistance in this film may rise as a result.

**Figure 5:**
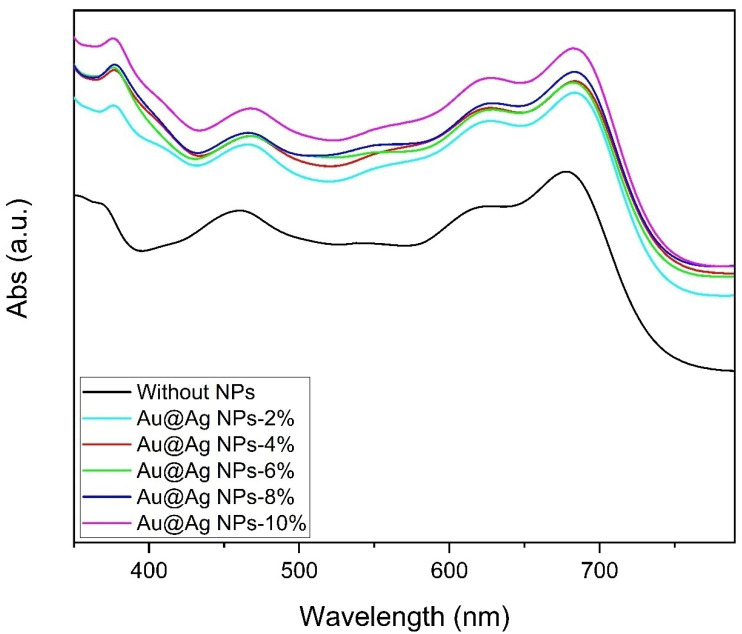
UV-Vis absorption profiles of the PSCs (ITO/PEDOT:PSS + Au@Ag NPs-14%/PTB7:PC_71_BM+ different amounts of Au@Ag NPs).

Moreover, the increased absorption of the active layer enhances the generation of e-h pairs in the PSCs. NPs play a crucial role in improving light absorption in the active layer. Contrarily, an increase in the volume ratio of NPs in the active layer could influence the electrical properties and serve as charge recombination centers. Figure 5 presents UV-Vis absorption spectra of multi-layer films of ITO/PEDOT:PSS + Au@Ag NPs-14%/ PTB7:PC71BM+ Au@Ag NPs exhibited increased absorptions in the range of 350–750 nm in comparison to the reference cell (with no NPs). It is notable that the higher improvement in the absorption in Au@Ag NPs-8% and Au@Ag NPs-10% compared to the other samples can be attributed to the increased concentration of Au@Ag embedded in PTB7:PC71BM.

**Figure 6:**
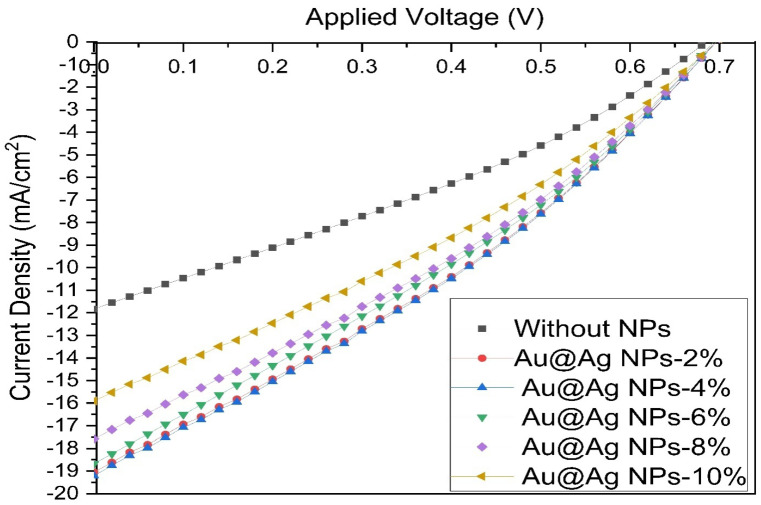
J-V curve of PSCs (ITO/PEDOT: PSS+Au@Ag NPs-14%/PTB7:PC71BM+ different amount of Au@Ag NPs).

**Figure 7:**
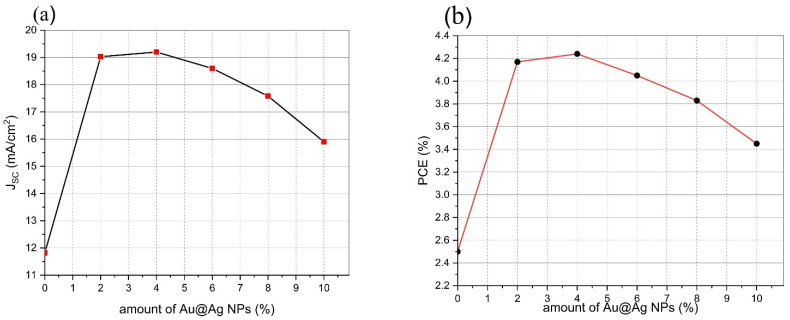
(**a**) J_sc_ and (**b**) PCE of solar cells with and without NPs.

Table 1 presents the electrical parameters J_sc_, V_oc_, FF, and PCE to elucidate the performance of the devices in the presence and absence of the Au@Ag NPs embedded into PEDOT:PSS and PTB7:PC71BM. J_sc_, V_oc_, FF, and efficiency of the reference device (devoid of Au NPs) were measured and calculated to be 11.82 mA/cm^2^, 685.8 mV, 30.9, and 2.5%, respectively. Figure 6 and Table 1 show that the J_sc_ values of devices were improved by incorporating the Au@Ag NPs in the devices. For ITO/PEDOT:PSS+Au@Ag NPs-14%/PTB7:PC71BM+Au@Ag NPs-4%/Al structure, J_sc_ and PCE of 19.20 mA/cm^2^ and 4.24% were obtained, respectively, which are markedly higher than those of the other devices fabricated in this study. This relatively high performance is attributable to the optimized value volume ratio of Au@Ag NPs incorporated in the buffer and active layers. The coupled effects of NPs at two dissimilar sites improve the positive influence of the LSPR, thus enhancing the absorption of the active layer. In addition, to compare the effects of different volume ratios of Au@Ag NPs embedded into PEDOT:PSS and PTB7:PC71BM on J_sc_ and PCE of the fabricated PSCs, J_sc_ and PCE curves of the samples are presented in Figure 7a,b, respectively. As observed in the J-V curve (Figure 6), the electrical characteristics of devices were not optimal at volume ratios of Au@Ag NPs-8% and Au@Ag NPs-10%, indicating parasitic absorption by the NPs themselves. Thus, raising the quantity of metallic NPs integrated into the active layer increased the direct interface between metallic NPs and the active layer, enhancing the charge recombination before reaching the donor–acceptor interface. Lastly, this study highlights the relative enhancement of PSCs by embedding Au@Ag NPs compared to the reference device (with no Au@Ag NPs) that is fabricated under similar synthesis conditions. Based on the results, PCE was improved by 69.6% from 2.50% to 4.24% of the device by embedding Au@Ag-4% into the active layer. As observed in Table 2, the device fabricated in this study displays significantly greater improvement in PCE compared with other results in the literature, which can be due to the dual plasmonic effects of embedding Au@AgNPs in the active and buffer layers.

## 4. Conclusions

This research demonstrates the effect of embedding durian-shaped Au@Ag NPs in two different layers (active layer and buffer layer) of PSCs on its PV performance. The ITO/PEDOT:PSS + Au@Ag NPs 14%/ PTB7:PC_71_BM + Au@Ag NPs 4%/Al structure showed a PCE improvement of 69.6% compared to the device fabricated without Au@Ag NPs. This improvement is attributable to embedding the PEDOT:PSS and PTB7:PC_71_BM devices with optimal volume ratios of Au@Ag NPs, combined with the LSPR effects of multi-positional Au@Ag NPs and their durian-shaped morphology. This approach to improving the performance of PSCs may pave the way for the development of low-cost, high-efficiency solar devices.

## Figures and Tables

**Table 1:** Electrical parameters of the PSCs with and without NPs.

No.	Au@Ag NPs in PEDOT:PSS	Au@Ag NPs in PTB7:PC71BM	J_sc_ (mA/cm^2^)	V_oc_ (mV)	J_max_ (mA/cm^2^)	V_max_ (mV)	FF (%)	PCE (%)
1	Without NPs	Without NPs	11.82	685.8	6.27	400	30.9	2.50
2	Au@AgNPs14%	Au@AgNPs-2%	19.03	697.3	10.44	400	31.5	4.17
3	Au@AgNPs-14%	Au@AgNPs-4%	19.20	699.0	10.60	400	31.6	4.24
4	Au@AgNPs-14%	Au@AgNPs-6%	18.60	694.0	10.13	400	31.4	4.05
5	Au@AgNPs-14%	Au@AgNPs-8%	17.58	696.1	9.57	400	31.3	3.83
6	Au@AgNPs-14%	Au@AgNPs-10%	15.90	698.4	8.63	400	31.1	3.45

**Table 2:** Summary of previous research on plasmonic MNPs and their corresponding PCE improvements.

No.	NPs	Location of MNPs	Relative Improvement in PCE (%)	Ref.
1	Ag nanospheres	In PEDOT:PSS	33	[51]
2	Au nanosphere	In PEDOT:PSS	24	[52]
3	Ag nanostructures	In ZnO and PTB7: PCBM	15	[37]
4	Au@PDA	On ITO	30	[30]
5	Ag-decahedron	In PEDOT:PSS	12	[28]
6	Au:Ag	On ITO	22.5	[31]
7	Au nanostars	In PEDOT:PSS	10.7	[53]
8	Au@Ag	In PEDOT:PSS and PTB7:PC71BM	69.6	-

## Data Availability

Not applicable.
